# Editorial: Interaction of Nanomaterials with the Immune System: Role in Nanosafety and Nanomedicine

**DOI:** 10.3389/fimmu.2017.01688

**Published:** 2017-11-28

**Authors:** Diana Boraschi, Lucio R. C. Castellano, Paola Italiani

**Affiliations:** ^1^Institute of Protein Biochemistry, National Research Council, Napoli, Italy; ^2^Federal University of Paraíba, João Pessoa, Brazil

**Keywords:** nanoparticles, immune system, innate immunity, inflammation, nanomedicine, nanovaccination

**Editorial on the Research Topic**

**Interaction of Nanomaterials with the Immune System: Role in Nanosafety and Nanomedicine**

In the past 20 years, engineered nanomaterials/nanoparticles (ENMs/ENPs) have become an increasingly common presence in our environment and everyday life. These new materials are included in a variety of products, such as solar cells, pigments and paints, tiles and masonry materials, and consumer products such as food packaging, cosmetics, and cigarettes. The long-term effects of exposure of human beings and environment to ENM are unknown, and concern is raising that ENM may have a detrimental impact on human health. In this perspective, it is of major importance assessing the interaction between ENM and the immune system, since in all living organisms the immune system is deputed to defending and maintaining the integrity of the body, and its failure is cause of damage and disease (1).

Engineered nanoparticles are being developed also for improving delivery of drugs, contrast agents, and antigens in therapeutic, diagnostic, and vaccination strategies. Such strategies aim at exploiting the unprecedented flexibility of design of ENP for tailoring them as efficient vehicles for delivering payloads to specific organs and locations, thereby increasing efficacy and minimizing side effects. Also in the case of nanomedicines, the interaction between the ENP and the host’s immune system is central, because nanomedicines need to avoid immune recognition and elimination. On the other hand, ENP can be used for targeting the immune system and stimulating its effectiveness in combating infections, cancer, and other diseases (1).

The Research Topic “Interaction of nanoparticles with the immune system: role in nanosafety and nanomedicine” intends to provide a snapshot of the latest trends in nanosafety and nanomedicine that take into consideration the interaction between ENM and the immune system.

Living organisms have encountered particles of different size since the very beginning of life on earth, thus the immune system of plants, invertebrates, and vertebrates is able to recognize particles (e.g., volcano ashes, viruses, and bacteria) and react to them by mounting a defensive response. ENM and ENP are new foreign agents for the immune system of both environmental species and human beings. Although we do not expect the immune system to have problems in dealing with ENM, similarly to other particles, nanoimmunosafety is becoming a matter of growing concern. In fact, ENM are produced in such a variety of chemical compositions, shapes, and sizes that predicting their interaction with immunity and the result of such interaction is very difficult. The review by Pallardy et al. specifically addresses this issue, underlying how and in which circumstances ENM and other nanoparticles can trigger immune responses that may represent a threat for human health. Other contributions address the interaction of specific EMN with the immune system. The group of Bengt Fadeel has examined graphene ENM and the mechanisms by which the innate immune system, in particular phagocytic cells, work for eliminating them (Mukherjee et al.). On the other hand, the review of the group of Victor Puntes underlines the need of a very accurate evaluation of the ENM characteristics in order to understand the variability of the effects on the immune system and the consequent possible safety concerns, taking as an example the case of cerium oxide ENP (Casals et al.). On the same line, two groups address more specifically the issue of endotoxin contamination in ENM as source of variability and misinterpretation of results: Li et al. review the topic and provide suggestions of how to test the presence of contamination, while Bianchi et al. show how titania ENP can adsorb LPS thereby becoming able to induce inflammation. A major issue in nanosafety is the capacity of ENM to activate complement and induce complement-mediated damage. Two contributions of the group of Dmitri Simberg and Moein Moghimi show how surface coating and surface functionalization with antibodies can significantly change the ability of iron oxide nanoworms to activate complement (Wang et al.; Wang et al.). Eventually, the group of Ken Ishii reviews the possibility of allergic reactions induced by skin exposure to EMN (Yoshioka et al.), while the group of Albert Duschl examines the role of ENM in type 2 immune responses and, together with the possible detrimental effects, proposes possible exploitation for therapeutic purposes (Himly et al.).

In nanomedicine, in order to deliver their therapeutic cargo, ENMs are designed so as to escape immune recognition and to penetrate defensive barriers, a strategy that increases targeted delivery and therapeutic efficacy. However, we should take great care in ensuring that nanomedicines do not interfere with immune surveillance and effectiveness, because a failing immune system will leave the body defenseless. On the other hand, nanomedical approaches can exploit the interaction of ENM with the immune system to obtain targeted delivery and targeted immune modulation. The review of La-Beck and Gabizon addresses the critical issue of efficacy of liposome-based anti-cancer therapies, which is most likely hampered by the interaction of administered liposomes with the host immune system. The group of Dan Peer provides important insights on the possibility of using lipid-based ENP, targeting the immune system, as immunotherapeutic agents that stimulate the host immune response against tumors (Mizrahy et al.). Along the very important strategy of targeting immune cells with ENM, Serkova shows how tumor-associated macrophages (TAM) can be specifically visualized with MRI using superparamagnetic iron oxide ENP, while the group of Biana Godin shows that drug-loaded ENP can actually modulate TAM polarization and shift it toward antitumor activation (Leonard et al.). Besides anti-cancer therapy, the use of ENM can be beneficial in other inflammation-based diseases such as neurodegenerative conditions, as suggested by the group of Paolo Decuzzi that shows decrease of amyloid β fibril-induced inflammation by delivering anti-inflammatory compounds to macrophages *via* ENP (Ameruoso et al.).

The last topic addressed is the use of ENM in vaccination. Indeed, particles have been included in vaccine formulations since ages, for increasing antigen persistence and for helping inducing and amplifying the protective adaptive immunity, but more recent studies plan to use ENM for a better control both of the antigen delivery and of the inflammation-based adjuvant effect (2). The group of Ana Paula Junquiera-Kipnis reviews the possibility of using metal ENP as adjuvants in vaccination (Moreira Marques Neto et al.), while the group of Alireza Haghparast designed polymeric ENP simultaneously carrying an antigen and two TLR agonists, which were very effective as vaccine in mice (Ebrahimian et al.). A very important issue, in designing ENP for inducing controlled NLRP3-dependent inflammation in vaccine adjuvanticity, is the issue addressed by the group of Yasuo Tsutsumi, i.e., that the control of ENP size is required in order to obtain the desired effect, as particles of the “wrong” size can lose their efficacy (Nishijima et al.). Finally, Italiani and Boraschi propose an original concept for the modulating immune responses with ENM, i.e., the personalized induction of innate memory, which in each individual subject can achieve controlled stimulation (as adjuvant in vaccination and in cancer therapy), or reduction of responses (for approaches in chronic inflammatory and autoimmune diseases).

In conclusion, this Research Topic provides a comprehensive vision of the state-of-the-art of the interaction of ENM with the immune system, its possible threats for human health and the very promising exploitation strategies. Numerous challenges are still outstanding, before we have a complete knowledge of such interaction, in particular in immunologically frail or compromised individuals, but the recent advancements make us believe that we will be soon able to use ENM effectively and safely.

## Author Contributions

DB and PI wrote the manuscript. LRCC assisted them.

## Conflict of Interest Statement

The authors declare that they have no financial or non-financial competing interests.
